# Mild cognitive impairment and microbiota: what is known and future perspectives

**DOI:** 10.3389/fmed.2024.1410246

**Published:** 2024-06-18

**Authors:** Antonella Gallo, Anna Maria Martone, Rosa Liperoti, Maria Camilla Cipriani, Francesca Ibba, Sara Camilli, Fiammetta Maria Rognoni, Francesco Landi, Massimo Montalto

**Affiliations:** ^1^Department of Geriatrics, Orthopedics and Rheumatology, Fondazione Policlinico Universitario “A. Gemelli”, IRCCS, Rome, Italy; ^2^Department of Geriatrics, Orthopedics and Rheumatology, Università Cattolica del Sacro Cuore, Rome, Italy

**Keywords:** cognitive, gut microbiota, gut-brain axis, mild cognitive impairment, probiotics

## Abstract

Mild cognitive impairment (MCI) is a heterogeneous condition definable as the intermediate clinical state between normal aging and dementia. As a pre-dementia condition, there is a recent growing interest in the identification of non-invasive markers able to predict the progression from MCI to a more advanced stage of the disease. Previous evidence showed the close link between gut microbiota and neurodegenerative diseases, such as Alzheimer's (AD) and Parkinson's disease (PD). Conversely, the actual relationship between gut microbiota and MCI is yet to be clarified. In this work, we provide an overview about the current knowledge regarding the role of gut microbiota in the context of MCI, also assessing the potential for microbiota-targeted therapies. Through the review of the most recent studies focusing on this topic, we found evidence of an increase of Bacteroidetes at phylum level and Bacteroides at genus level in MCI subjects with respect to healthy controls and patients with AD. Despite such initial evidence, the definitive identification of a typical microbiota profile associated with MCI is still far from being achieved. These preliminary results, however, are growingly encouraging research on the role of gut microbiota modulation in improving the cognitive status of pre-dementia subjects. To date, few studies evaluated the role of probiotics in MCI subjects, and they showed favorable results, although still biased by small sample size, heterogeneity of study design and short follow-up.

## 1 Introduction

In the latest years, growing evidence showed the close link between neurodegenerative diseases and gut microbiota.

In particular, the so-called “microbiota-gut-brain axis” identified a bidirectional system characterized by a constant interplay between gut and brain with a consequent influence between each other (1, 2). It is therefore not surprising that intestinal “dysbiosis”, a condition of imbalance in the composition and function of the bacterial flora, is implicated in the pathogenesis and progression of some neurological diseases, as for many other conditions (3). While this correlation is better defined in conditions such as Parkinson's disease (PD) and Alzheimer's disease (AD) (3–5), only few studies focused on the possible role of gut microbiota in Mild Cognitive Impairment (MCI). MCI represents a heterogeneous condition, sometimes identifiable as a pre-dementia stage (6).

It would therefore be desirable to identify specific markers able to predict the progression from MCI to overt dementia, and, even better, to evaluate their role as potential targets for therapeutic interventions.

The aim of this narrative review is to provide an overview about the role of intestinal microbiota in the context of MCI, from pathogenesis to disease progression, including identification of potential microbiota-targeted therapies. Firstly, we conducted a literature search on PubMed using the keywords: “mild cognitive impairment”, “cognition” and “gut microbiota” “probiotics” “gut-brain axis”. Once all duplicates were removed, all pertinent and most relevant articles published in English between 2001 and 2024 were finally included in this review (original articles, reviews, meta-analysis, case reports).

## 2 Mild cognitive impairment

Mild Cognitive Impairment (MCI) is a heterogeneous condition definable as the intermediate clinical state between normal brain aging and dementia (6, 7). The affected individuals complain of greater cognitive deficits than it would be expected for their age and education, but not significantly interfering with their daily activities (6, 8). MCI is common in older people, and its prevalence rises with age, reaching approximately 25% in subjects aged between 80 and 85 years (9). MCI can be also defined as an at-risk status for dementia. In fact, subjects with MCI are approximately three times more likely to develop dementia over 2–5 years compared with age-matched controls (9). Most studies report conversion rates from MCI to dementia between 10 and 20% per year (10). MCI with prevalent memory impairment (amnestic MCI) is the most prevalent neuropsychological subtype and also the one with the highest probability to progress to dementia due to Alzheimer's disease (11). It also true that a certain proportion of subjects with MCI, up to 30% based on different studies, improve, even to normal, over a one- to three-year time (12).

The variability in the MCI progression trajectory reflects the heterogeneity of the condition that may be related to different underlying brain pathological processes. In fact, although more frequently associated with Alzheimer's disease and neurodegenerative damage, MCI can be also secondary to other underlying pathologies (i.e., vascular, metabolic, traumatic, psychiatric) (8). For example, a quite frequent condition is MCI due to a vascular damage (i.e. ischemia, infarction, hemorrhage) in brain parenchyma (13). This condition, known as vascular cognitive impairment, may evolve into vascular dementia and represents one of the main causes of dementia in the western world following Alzheimer's disease (13). Moreover, MCI in Parkinson's disease (PD—MCI) presents itself a distict pathological and clinical entity, with variable clinical presentation (14). Finally, treatable conditions psychiatric diseases (particularly depression), metabolic disorders (hypothyroidism, vitamin B12 deficiency) and side effects of medications, may present with clinical features of MCI (9).

To date, criteria for the diagnosis of MCI are based on clinical judgement and neuropsychological assessment. A workgroup on diagnostic guidelines from the National Institute on Aging and the Alzheimer's Association defined the following core clinical features that indicate MCI due to Alzheimer disease (AD): (a) a change in cognition reported by the person or an informant or observed by clinician; (b) evidence of impairment in one or more cognitive domains, typically including memory, at neuropsychological assessment; (c) preserved independence in daily life functional abilities; (d) absence of dementia (15). Other possibile etiologies of MCI including other neurodegenerative, vascular, metabolic, psychiatric conditions should be also evaluated if clinically suspected. In the fifth edition of the Diagnostic and Statistical Manual for Mental Disorders (DSM-5) the term mild neurocognitive disorder (NCD) refers to MCI and diagnostic criteria to define it include reported cognitive impairment and related concern by the person, an informant or observed by clinician, mild cognitive impairment at the neuropsychological assessment that do not interfere with functional abilities, absence of delirium and psychiatric conditions such as major depression and schizophrenia (16).

Imaging tests (Magnetic Resonance Imaging, Positron Emission Tomography) and biomarkers (beta amyloid, total and phosphorylated tau) have been proposed with the only purpose of clarifying the etiology of MCI and predict progression in research setting (15).

Current guidelines recommend to control modifiable risk factors (i.e. high blood pressure, depression, sleep apnea), promote physical exercise and cognitive training (9, 17). To date, no medications have been approved for the treatment of MCI in Europe. Nonetheless, MCI subjects, especially those with Alzheimer pathology, are considered potential target for innovative treatments currently approved by the FDA in US such as monoclonal antibody directed against amyloid.

Finally, growing research efforts are directed to the identification of non-invasive markers that may characterize different clinical subtypes of MCI and predict progression, thus allowing the selection of those subjects who are at highest risk of developing dementia.

## 3 The gut–brain axis

In recent decades, there has been increasing awareness of how the intestinal microbiota plays a role in the regulation and homeostasis of most physiological processes, including nutritional metabolism, resistance to infections and the development and functioning of the immune and nervous systems (2).

The intestinal microbiota is the set of different species of symbiotic and commensal microorganisms located in the gastro-intestinal tract, mostly in the ileum and colon (18). Microorganisms are mainly bacteria, but also archaea, viruses, fungi and protozoa, and coexist in a mutualistic relationship with the host (18). Gene sequencing techniques have identified 100 billion bacteria, at least 1,000 species and more than 7,000 strains, populating the intestinal microbiota and making it approximately 150 times more genetically diverse than the human body (19). Four main phyla are the most frequently recognized and they include Firmicutes, Bacteroides, Proteobacteria and Actinobacteria. Among them, Firmicutes and Bacteroides represent the majority (approximately 90%) (20). The process of bacterial colonization begins at birth and continues throughout life, and it is influenced by various factors, such as age, diet, region of origin, gender and genetics (21, 22).

The gut microbiota plays pivotal functions for the wellness of the individual; therefore, it is not surprising how an alteration in its composition, which leads to a condition of “dysbiosis”, can be a sign of latent pathologies and predisposition to others. It has a defensive—immune role, contributing to the formation of a protective barrier against pathogens and promoting the production of immunomodulatory substances that regulate the body's immune response (22). It is involved in metabolic processes, influencing the body's energy balance, and in digestive processes, contributing to the synthesis of vitamins (22). Finally, the microbiota influences the maturation and development of the central nervous system, playing a fundamental role in the so-called “gut-brain axis”, with a constant bidirectional communication between the gastrointestinal tract and the brain (2).

To date, three main ways of communication have been recognized between the gut and the brain: (1) direct neuronal messages via the vagus nerve; (2) endocrine secretion conveyed by gut hormones; (3) immune messages conveyed by cytokines (22). All of them are mediated by gut microbiota. In particular, intestinal bacteria are able to produce short-chain fatty acids (acetate, butyrate) from dietary components, playing a fundamental role in modulating the integrity of the blood-brain barrier and a protective role in brain function (18). In fact, butyrate may enhance gut barrier and blood–brain barrier (BBB) by increasing expression of tight-junction proteins and producing antimicrobial peptides (23). Meanwhile, it can regulate expression of brain-derived neurotrophic factor (BDNF) and N-methyl-D-aspartic acid (NMDA) receptors, promote neurogenesis, and participate in the formation of synaptic plasticity (24).

Furthermore, the different bacteria that populate the gut microbiota are implicated in the catabolism of amino acids and in the consequent production of neurotransmitters, involved in cognitive and behavioral functions (18). Species of the genera Lactobacillus and Bifidobacterium, for example, produce gamma-aminobutyric acid, the main inhibitory neurotransmitter of our organism, through the metabolism of glutamate (25, 26). Similarly, the microbiota is involved in the correct synthesis of serotonin, regulating the levels of its precursor, tryptophan (27). Finally, mechanical, chemical and hormonal stimuli coming from the microbiota act at the level of the receptors of the vagal afferents and efferents present at the level of the gastrointestinal tract and implicated in the transmission of information to the central nervous system. They are therefore implicated in the digestion signaling pathway, contributing to gastrointestinal motility, feelings of hunger and eating behaviors (28).

## 4 Gut microbiota in mild cognitive impairment

The interest of experts in the field has growingly moved toward the study of gut microbiota and its correlation with the development and progression of various pathologies, including neurodegenerative diseases.

The microbiota composition has been extensively evaluated in subjects affected by AD, founding some peculiarities in comparison to healthy controls (HC). In particular, most studies agreed with a significant reduction of *Bacteroidetes* (29–31) and *Firmicutes* (29, 30, 32–35) in the gut microbial composition of AD patients compared to HC. On the other hand, an increase of *Proteobacteria, Actinobacteria* and *Verrucomicrobia* was reported in AD (29–31).

The knowledge expanded also through Parkinson disease (PD) and its correlation between cognitive impairment and microbiota. Different studies found an association between a worse clinical profile with higher frequencies cognitive impairment and a reduction in some families and genera of the phyla *Firmicutes* and *Bacteroidetes* in PD subjects with respect to HC (36, 37).

Recently, attention switched to the study of pre-dementia stages, notably MCI, mainly to identify non-invasive markers able to predict the progression to overt dementia. However, evidence regarding gut microbiota in MCI is still sparse and the potential efficacy of microbiota-targeted therapies in this condition is yet to be clarified.

To date, only a total of 14 studies and two systematic reviews and meta-analyses (31–35, 38–48) had focused on the correlation between gut microbiota composition and MCI. Most of them made a comparison between MCI and AD/PD patients or between MCI and healthy controls.

Results from these studies are not definitive and quite controversial (Table 1).

**Table 1:** Gut microbiota composition studies in patients with mild cognitive impairment.

**References**	**Study design**	**Study population**	**Country**	**Gut microbiota profile in MCI**
Liu et al. (33)	Observational	32 MCI; 33 AD; 32 HC	China	↑*Gammaproteobacteria, Enterobacteriales, Enterobacteriaceae* (HC < MCI < AD)
Li et al. (31)	Observational	30 MCI; 30 AD; 30 HC	China	↑*Dorea, Lactobacillus, Streptococcus, Bifidobacterium, Blautia, Escherichia* (MCI>HC, no differences with AD) ↓*Alistipes, Bacteroides, Parabacteroides, Sutterella, Paraprevotella* (MCI < HC, no differences with AD)
Saji et al. (38)	Observational	61 MCI; 21 HC	Japan	↑*Bacteroidetes, Bacteroides* (MCI>HC)
Ren et al. (39)	Observational	13 PD-MCI; 14 PD-NC	China	↑*Alistipes, Barnesiella, Butyricimonas, Odoribacter* (PD-MCI>PD-NC) ↓*Blautia Ruminococcus* (PD-MCI < PD-NC)
Wu et al. (32)	Observational	22 MCI; 27 AD; 28 HC	China	↑*Bacteroides* (MCI>HC and AD)
Pan et al. (40)	Observational	22 MCI; 26 HC	China	↓*Bacteroides salyersiae* (MCI < HC)
Wanapaisan et al. (34)	Observational	12 MCI; 20 AD; 20 HC	Thailand	↑*Bacteroidetes, Enterobacteriaceae* (MCI>HC, not significant differences with AD) ↓*Clostridiaceae* (MCI < HC, not significant differences with AD)
Sheng et al. (41)	Observational	11 MCI; 11 AD; 66 HC	China	↓*Firmicutes* (MCI < HC, not significant differences with AD)
Zhu et al. (42)	Observational	125 MCI; 83 AD; 94 HC	China	↑*Erysipelatoclostridiaceae, Erysipelotrichales, Patescibacteria, Saccharimonadales*, and *Saccharimonadia* (MCI>HC, not significant differences with AD)
Aljumaah et al. (43)	RCT PC	44 MCI; 125 HC	USA	↑*Prevotella* (MCI>HC)
Pei et al. (44)	Observational	38 MCI; 45 AD; 35 HC	China	↑*Bacteroidetes* (MCI>HC>AD) ↑*Bacteroides, Alistipes, Roseburia, Tyzzerella* 4 (MCI>HC and AD) ↑*Allisonella, Faecalibacterium, Flavonifractor, Fusicatenibacter, Lachnospiraceae* ND3007 group, *Prevotella* 7, *Prevotella* 9 (MCI>AD)
Kim et al. (45)	Observational	40 MCI; 40 HC	Korea	↑*Bacteroides* (MCI>HC)
McLeod et al. (46)	Observational	30 MCI; 30 HC	USA	↑*Bacteroides* (MCI>HC)
Sánchez-Tapia et al. (47)	Observational	32 MCI; 42 HC; 13 D	Mexico	↑*Prevotella stercorea* (MCI>HC>D)
Chen et al. (35)	Meta-analysis	2643 MCI/AD; 2336 HC	-	↓*Firmicutes* (MCI < HC) ↑*Bacteroidetes* (MCI>HC; not significant difference between AD and HC) ↓*Lactobacillus* (MCI < HC)
Jemimah et al. (48)	Meta-analysis	241 MCI; 438 AD; 632 HC	-	Not significant differences between MCI, AD and HC

MCI, mild cognitive impairment; AD, Alzheimer's disease; HC, health control; PD-MCI, Parkinson's disease with mild cognitive impairment; PD-NC, Parkinson's disease with normal cognitive; RCT PC, randomized controlled trial placebo-controlled; D, dementia.

### 4.1 Composition of gut microbiota in MCI compared to AD patients and healthy controls

Most studies comparing microbiota composition between AD and MCI patients testified an increase of *Bacteroidetes* at phylum level and *Bacteroides* at genus level in MCI with respect to healthy controls (HC) and AD patients.

In particular, Wu et al. identified an altered microbiota composition in subjects with amnestic mild cognitive impairment (aMCI) and AD. They recruited 77 individuals (22 aMCI, 27 AD, and 28 HC). Firstly, analyzing metabolomic profiling, they found fecal indole-3-pyruvic acid (IPYA), a metabolite from tryptophan, progressively enriched from aMCI and AD compared with HC while five SCFAs (including formic acid, acetic acid, propanoic acid, 2-methylbutyric acid, and isovaleric acid) progressively decreased from aMCI to AD patients. Then, they compared SCFA-producing bacteria between HC, aMCI, and AD patients. They found that Clostridia, with the capability of producing SCFAs like acetate, propionate, butyrate and its phylum *Firmicutes (*as well as the *Clostridiales, Ruminococcaceae, Ruminococcus* at order, family and genus levels), were significantly decreased in AD patients compared to aMCI and HC and, while the propionate-producing *Bacteroides* was increased in aMCI patients compared to AD patients and HC (32).

Consistently with these results, Liu et al. found a significant reduction of phylum *Firmicutes* in AD, whereas *Gammaproteobacteria, Enterobacteriales* and *Enterobacteriaceae* showed a progressive enriched prevalence from HC to aMCI and AD patients (33).

Recently, Chen et al., in a systematic review and meta-analysis, confirmed that the amount of *Firmicutes* at the phylum level was significantly lower in patients with AD and MCI compared to HC. They also confirmed that the amount of *Bacteroidetes* at the phylum level, commonly reduced in AD patients, was instead significantly higher in patients with MCI subjects compared to HC. Additionally, they demonstrated an increasing trend for *Enterobacteriaceae* and a decreasing trend for *Ruminococcaceae, Lachnospiraceae* and *Lactobacillus* in AD patients, with an early decreasing trend of *Lactobacillus* also in MCI patients (35).

In the same years, Pei et al. conducted a cross-sectional study, recruiting a total of 118 subjects (45 AD, 38 MCI, and 35 HC). Also in this study, MCI group had higher abundance of *Bacteroidetes* at phylum-level compared to the other two groups; at genus-level, MCI group showed higher abundance of *Bacteroides* with respect to the other two groups, together with *Alistipes, Roseburia, and Tyzzerella 4*. They also found more *Allisonella, Faecalibacterium, Flavonifractor, Fusicatenibacter, Lachnospiraceae ND3007 group, Prevotella 7, and Prevotella 9* in MCI subjects compared to AD group. Interestingly, through a correlation analysis, the authors found that *Bacteroides* and *Faecalibacterium* were both negatively correlated with content of endotoxin and positively correlated with scores of the cognitive assessment scales, suggesting that these bacteria were positively involved in preservation of intestinal barrier integrity and cognition status maybe through the protective role of their SCFAs metabolites (44).

Conversely, other studies didn't find any significant difference in gut microbiota composition between AD and MCI subjects, while they have confirmed a different gut profile among these two conditions and HC (31, 34, 41, 42). Wanapaisan et al. reported an increase of *Bacteroidetes* in both MCI and AD subjects (34), differently therefore from the majority of studies showing a reduction of *Bacteroidetes* in advanced disease stage. Also, Jemimah et al. found quiet and not statistically significant changes in MCI subjects compared to AD. However, they reported a trend toward a moderate increase for *Firmicutes* phylum and a tendency toward a moderate decrease for *Bacteroidetes* phylum with respect to HC (48).

Few studies compared gut microbiota composition only between MCI and healthy controls, most of them confirming the increase of *Bacteroidetes* (38) at phylum level and *Bacteroides* at genus level (38, 45, 46) in MCI subjects compared to HC. Kim et al. recruited 80 patients (40 MCI, 40 HC) in Korea, confirming at genus level the increase of *Bacteroides* in the samples of MCI subjects, according to previous studies (45). Moreover, Aljumaah et al. identified *Prevotella* (phylum *Bacteroidetes*) as significantly more prevalent in MCI compared to cognitively intact subjects. They also found an amount of some bacteria in most of HC, such as *Bifidobacterium longum, Bifidobacterium breve*, and *Faecalibacterium prausnitzii*, otherwise not present in the group with MCI, thus suggesting their protective role in the development of cognitive decline (43). Similarly, Sanchez-Tapia et al. found at species level an increase in *Prevotella stercorea* (phylum *Bacteroidetes*) in subjects with MCI, with respect to the patients without cognitive impairment and those with dementia (47).

On the other side, Pan et al. in a study among 48 participants (22 MCI and 26 HC) found that *Bacteroides salyersiae* (phylum *Bacteroidetes*) were decreased in the MCI group (40).

### 4.2 Composition of gut microbiota in MCI compared to PD

Although there is a general consensus about the relationship between gut microbiota and cognitive impairment in PD (36, 37, 49), there is also a study focused on comparison between gut microbiota composition in MCI and PD patients with a normal cognitive status. Ren et al. analyzed gut microbiota in a cohort of 27 patients affected by PD, showing that gut microbiota composition of patients with PD and MCI was significantly different compared to patients affected by PD but without a cognitive impairment. In particular, the MCI-PD group showed enriched, at genus level, of *Alistipes, Barnesiella, Butyricimonas*, and *Odoribacter* and decreased abundance of *Blautia* and *Ruminococcus* (39).

## 5 Therapeutic interventions by microbiota modulation in mild cognitive impairment

As MCI represents a high-risk status for developing dementia and it also constitutes the prodromal stage of various neurological disorders, understanding the underlying mechanisms and better characterizing this condition appears crucial to identify potential targets of preventive and therapeutic strategies. Indeed, a meta-analysis conducted by Lv et al. shows how probiotic supplementation determines higher improvement in cognitive status in patients with cognitive decline than in healthy individuals (50). However, the studies included in this meta-analysis focused on conditions with secondary cognitive decline, such as depressive syndrome and hepatic encephalopathy.

In addition, several studies focused on evaluating the effectiveness of probiotic supplementation in patients with established Alzheimer's disease (51, 52), while few studies included subjects with MCI. Given the established correlation between changes in microbiota composition and the presence of early cognitive decline, some works have evaluated the possibility of intervention in this area (Table 2). In recent years, the majority of studies have focused on assessing the effectiveness of intervention strategies based on the administration of probiotics.

**Table 2:** Therapeutic intervention studies in patients with mild cognitive impairment.

**References**	**Study design; study population; duration**	**Intervention**	**Methods**	**Main findings**	**Limits**
Hwang et al. (53)	DBRCT PC; 100 MCI; 12 weeks	*50 L..plantarum C29 vs* 50 placebo	CNT, serum BDNF levels, Gut microbiota analysis	↑ in CNT scores in the probiotic group, ↑ BDNF levels in the probiotic group, ↑ in *Lactobacillus* spp. in the probiotic group	Brief follow-up duration Only specific cognitive domains considered No correlation between BDNF serum levels, cognitive impairment and probiotic No comparison between MCI and HC
Kobayashi et al. (54)	DBRCT PC; 121 MCI; 12 weeks	*61 B.breve A1 vs* 60 placebo	Japanese version of the RBANS, MMSE	No significant difference in cognitive outcomes except for patients with low RBANS score at baseline (↑ in immediate and delayed memory fields and MMSE compared to placebo)	Brief follow-up duration Different RBANS at baseline No comparison between MCI and HC No microbiome analysis
Nagpal et al. (55)	DBRCT crossover; 11 MCI/6 HC; 6 weeks	MMKD *vs* AHAD	Gut microbiota analysis, measurement of FOAL, CSF analysys	in MCI patients on MMKD: ↓ in the *Actinobacteria* phylum, *Bifidobacteriaceae* family, *Bifidobacterium* genus and fecal lactate levels, ΔAß42: -PosC: family *Rikenellaeae*; genus *Parabacteroides*-NegC: phylum *Tenericutes*; family *Enterobacteriaceae* Δtau-p181: -PosC: genus *Sutterella -*NegC: class *Mollicutes*	Small sample size Brief follow-up duration Absence of cognitive assessment
Sanborn et al. (56)	DBRCT PC; 42 MCI/103 HC; 12 weeks	76 *L.rhamnosus GG vs* 69 placebo	NIH Toolbox for cognitive assessment	Cognitive improvement of MCI in the probiotic group	Brief follow-up duration Patients' self-reports for diseases No microbiome analysis
Aljumaah et al. (43)	DBRCT PC; 44 MCI/125 HC; 12 weeks	86 *L.rhamnosus GG vs* 83 placebo	NIH Toolbox for cognitive assessment, Gut microbiota analysis	↓ genus *Prevotella* and *Dehalobacterium* correlated with cognitive improvement of MCI in the probiotic group	Brief follow-up duration Patients' self-reports for diseases
Asaoka et al. (57)	DBRCT PC; 115 MCI; 24 weeks	55 *B.breve A1 vs* 60 placebo	Japanese version of ADAS-Jcog, MMSE, VSRAD, gut microbiota analysis	↑ in “orientation” ADAS-Jcog in the probiotic group ↑ in “orientation in time” and “writing” MMSE in the probiotic group No significant difference in microbiome composition	Brief follow-up duration No comparison between MCI and HC
Fei et al. (58)	RCT PC; 42 MCI; 12 weeks	21 Mix of *Lactobacilli* and *Bidifobacteria vs* 21 placebo	MoCA, MMSE, PSQI, GSRS, serum BDNF levels, gut microbiota analysis	↑ “attention”, “calculation”, and “recall” MMSE subscales, ↑ in recall, visuospatial, and executive MoCA scores, ↑ PSQI, GSRS scores, ↑ BDNF levels, ↑ relative abundance of *Bacteroidetes* in the probiotic group	Small sample size Brief follow-up duration No comparison between MCI and HC

↑, improvement/increase; ↓, reduction; Δ, changes; ADAS-Jcog, Alzheimer's Disease Assessment Scale; AHAD, American Heart Association diet; CNT, Computerized Neurocognitive Test; CSF, cerebrospinal fluid; DBRCL PC, Double-blind randomized clinical trial placebo controlled; DBRCL, Double-blind randomized clinical trial; FOAL, fecal organic acid levels (acetate, propionate, and butyrate); GSRS, Gastrointestinal Symptom Rating Scale; HC, healthy control; MMKD, Modified Mediterranean ketogenic diet; MMSE, Mini-Mental State Examination; MoCA, Montreal Cognitive Assessment Scale; NegC, negatively correlation; PosC, positively correlation; PSQI, Pittsburgh Sleep Quality Index; RBANS, Repeatable Battery for the Assessment of Neuropsychological Status; RCT, randomized controlled trial; VSRAD, Voxel-based Specific Regional Analysis System for Alzheimer's disease.

A double-blind, randomized study conducted by Sanborn et. al investigated the effect of administration of *Lactobacillus rhamnosus* GG (LGG) in patients with MCI and healthy individuals, with cognitive function assessed using the NIH Toolbox for the Assessment of Neurological and Behavioral Function and Cognition scales. Subjects with a previous history of neurological or psychiatric disorder were excluded from the study, as well as persons taking antibiotics, proton pump inhibitors, other prebiotic or probiotic supplements. Severe hepatic, renal, cardiological, gastrointestinal diseases and immunosoppression were also exclusion criteria. The study involved a total of 145 participants (HC *n* = 103, MCI *n* = 42) aged between 52 and 75 years (mean 64.4) who were randomized to placebo or the supplementation (probiotic n = 76, placebo n = 69). After 3 months, the change in the cognitive status was assessed. Among MCI subjects, the intervention resulted in improved cognitive status for subjects who received the supplementation with probiotic compared to those who were given placebo. It is interesting to note that healthy subjects undergoing probiotic supplementation did not experience any benefit from the intervention (56). The study was limited by the brief follow-up duration and the reliance on participants' self-reports. Additionally, Aljumaah et al. conducted a microbiome analysis on patients examined in the same study, at baseline and at 3 months. The results showed that the reduction in the relative abundance of the genus Prevotella and Dehalobacterium after LGG supplementation correlated with an improvement in cognitive scores in subjects with MCI (43).

Another study focused on the impact of supplementation with *Lactobacillus plantarum* C29-fermented soybean through a multi-center study with a 12-week observation period (53). The sample consisted of 100 individuals diagnosed with MCI according to the Diagnostic and Statistical Manual of Mental Disorders (DSM-5), who were randomly assigned to either the placebo or the supplementation group (placebo *n* = 50, probiotic *n* = 50). Exclusion criteria included the presence of known pathologies causing cognitive dysfunction, the use of supplements in the 2 weeks prior to the study, the presence of relevant psychiatric or clinical diseases, and patients on pharmacological therapy that could have altered cognitive status (e.g., antipsychotics or antidepressants). This study not only showed an improvement in cognitive function scores (assessed through computerized neurocognitive function tests) in participants taking the supplement but also an increase in serum levels of brain-derived neurotrophic factor (BDNF) in this subgroup. In this study, a microbiota analysis was also conducted on 92 of the 100 enrolled subjects (45 treated with the probiotic and 47 patients in the placebo group) at both baseline and follow-up, using DNA analysis on fecal samples. Findings demonstrated an increase in Lactobacillus spp. within the probiotic group compared to those in the placebo group, while Bifidobacterium spp. and Clostridium spp. did not show significant changes in either the control or intervention group. The study limitations in this work include the confined observation period of 12 weeks, the examination restricted to only a subset of specific cognitive domains (attention, working memory, and verbal memory), and the absence of a known correlation between BDNF values, cognitive improvement, and the chosen probiotic.

Kobayashi et al. assessed the effect of supplementation with *Bifidobacterium breve* A1 in patients with cognitive dysfunction, particularly affected in the domain of memory. The randomized, double-blind, placebo-controlled study involved 121 participants aged 50 to 80 years with a Mini Mental State Examination (MMSE) score of 22–27. The selected subjects did not have a diagnosis of dementia, psychiatric disorders, or severe medical conditions, nor did they have a history of major surgery in the digestive tract. They also had no history of drug or alcohol abuse and did not adhere to a specific diet or exhibit an irregular lifestyle. The cognitive assessment was conducted using the Japanese version of the Repeatable Battery for the Assessment of Neuropsychological Status (RBANS) and the MMSE. Results showed that the two groups (placebo *n* = 60, supplement n=61) did not exhibit significant differences in terms of changes in cognitive scores at 12 weeks compared to baseline. However, when the population was further divided into two subgroups, those with a low RBANS score at baseline (< 41 points) treated with probiotic demonstrated improvement in the fields of immediate and delayed memory compared to the low-score subgroup who received the placebo. Additionally, the total MMSE score was significantly improved in the *B. breve A1* group at 12 weeks vs. the placebo group. This difference suggests that probiotic supplementation may provide more benefit the earlier the detection of initial cognitive decline but it could not be beneficial in subjects who already show a nearly intact cognitive function before the treatment. Similarly to the other studies mentioned, in this work, a limitation is certainly imposed by the short observation period. The significant difference in the RBANS score at baseline between the two groups is also a study limitation (and for this reason, stratification into two subgroups based on this score was necessary). Moreover, the absence of prior studies comparing the effectiveness of B. breve A1 in subjects with MCI and healthy individuals is another limitation that could have provided valuable insights into clarifying the observed probiotic efficacy in individuals with a low RBANS score at baseline (54).

The impact of *Bifidobacterium breve A1* was also assessed in a 2022 study conducted by Asaoka et al., where 115 subjects aged between 65 and 88 with MCI received either the probiotic (*n* = 55) or a placebo (*n* = 60) with a follow-up at 24 weeks. Cognitive scales utilized in the study included the Japanese version of Alzheimer's Disease Assessment Scale (ADAS-Jcog) and MMSE. Patients treated with the probiotic had an improvement in the “orientation” subscale of ADAS-Jcog at 24 weeks. The study also revealed that, when the population was further divided into two groups based on the MMSE score at baseline, those with a lower MMSE (< 25) showed an improvement in the MMSE “orientation in time” and “writing” subscales. Interestingly, patients receiving the placebo exhibited worsening cerebral atrophy at 24 weeks, measured by MRI brain using the Voxel-based Specific Regional Analysis System for Alzheimer's disease (VSRAD). This was not occurring in the supplementation group. The microbiota analysis also revealed no significant difference in overall composition between the two groups. This suggests that the benefits derived from probiotic supplementation may not be solely attributed to quantitative or qualitative changes in the gut microbiota but rather to mechanisms that are yet to be defined (57).

Finally, a more recent study conducted by Fei et al. (58) evaluated the administration of a probiotic mix, primarily consisting of *Lactobacilli and Bifidobacteria*, for 12 weeks in subjects over the age of 60 with MCI according to Peterson criteria. A total of 42 participants were randomly assigned to either the placebo or probiotic group in a controlled randomized manner. The scales used for cognitive assessment were the MMSE and the Montreal Cognitive Assessment Scale (MoCA). In addition to cognitive evaluations, the Pittsburgh Sleep Quality Index (PSQI) and the Gastrointestinal Symptom Rating Scale (GSRS) were calculated. These scales were assessed at baseline and at 12 weeks, along with the determination of serum Brain-Derived Neurotrophic Factor (BDNF) levels. The result of this study showed an improvement in the MMSE score at 12 weeks in patients who took the probiotic, particularly in the domains of attention, calculation, and recall. As for the MoCA assessment, an improvement was observed in recall, visuospatial, and executive scores compared to the placebo group. Gut microbiota analysis revealed an increase in the relative abundance of *Bacteroidetes* after the probiotic intervention. Probiotic supplementation also demonstrated an improvement in sleep quality and gastrointestinal symptoms, with an increase in BDNF levels. This study was primarily limited by the small number of participants and the absence of a control group including healthy individuals.

The studies regarding dietary interventions in patients with MCI are limited, and the few that exist do not provide long-term analysis but are constrained by short observation periods. Many studies have focused on assessing the detrimental effects of a high-fat diet, such as the Western diet (59, 60), while potential benefits were reported with the Mediterranean diet (61–63). Combining the well-known beneficial effects of the Mediterranean diet and the demonstrated benefits of the ketogenic diet on certain neurological conditions, such as refractory epilepsy, Nagpal et al. conducted a study to assess the effectiveness of the so-called “modified Mediterranean ketogenic diet” (MMKD) (55). This diet is based on the ketogenic model but incorporates a higher intake of carbohydrates and fats from plant sources, fruits, and vegetables, as typically seen in the Mediterranean diet. The study involved 17 participants with an average age of 64.6, including 6 healthy subjects and 11 diagnosed with MCI based on Alzheimer's Disease Neuroimaging Initiative 2 (ADNI-2) criteria. Participants were randomly assigned to either the MMKD or the diet defined by the American Heart Association (a diet characterized by a low-fat percentage and higher carbohydrate consumption) in a randomized, double-blind, crossover manner. Microbiota analysis, measurement of organic acid levels (acetate, propionate, and butyrate) in feces, and concentration of Aβ-42, Aβ-40, tau, and phospho-tau (tau-p181) levels in cerebrospinal fluid were conducted. Reassessment 6 weeks after the diet showed no significant impact on the overall microbiome, but there was a reduction in the Actinobacteria phylum, Bifidobacteriaceae family, and Bifidobacterium genus in MCI subjects undergoing the MMKD. At the same time, these subjects exhibited a modulation with reduction in fecal lactate levels compared to healthy patients. Analysis of cerebrospinal fluid biomarkers showed that phylum *Tenericutes* and family *Enterobacteriaceae* were negatively correlated with changes of Aβ-42, while Mollicutes was negatively correlated with tau-p181 in MCI patients after MMKD. The study was limited by a higher representation of White American ethnicity and female gender (both ethnicity and gender are factors influencing the composition of the microbiota), the brief observation period, and the small sample size. Lastly, this study primarily investigated how a specific diet could differently modulate the microbiota composition in subjects with MCI, but the effect of the intervention on cognitive improvement was not investigated.

## 6 Discussion

In the last few years, the gut microbiota has represented an interesting topic to be explored due to its correlation with the development and progression of various conditions, including neurodegenerative diseases. In the context of dementia, the gut microbiota composition has been extensively studied in AD and PD, showing peculiar differences from healthy controls, thus leading to suppose that modulating these alterations by use of probiotics may represent a therapeutic weapon (42). Recently, the interest of the scientific community switched to the study of pre-dementia condition, notably known as MCI, encouraging search for peculiar findings and non-invasive markers able to predict progression to dementia. However, intestinal microbial research regarding MCI remains limited and, most of all, lacks a unique consensus.

The actual state of art confirms that it is difficult to find a specific microbiota profile that characterizes MCI. However, studies indicate that there are some differences between microbial characteristics of MCI and proper dementia, such as the advanced stage of AD and PD. In general, most of these studies agreed that there is an increase of *Bacteroidetes* at phylum level and *Bacteroides* at genus level in MCI with respect both to healthy controls (HC) and AD patients. Anyway, not all studies agree with this statement and show other differences among species and genera of bacteria. This is probably due to the great heterogeneity of MCI populations and to the lack of adjustment of various factors, such as comorbidities and medications, which might affect the gut microbial composition. Moreover, their heterogeneity might be explained by the various cohorts of patients, analyzed in studies conducted in different world's areas, thus supporting the notion that region, diet and lifestyle may have a considerable influence on the gut microbiota composition and, maybe, in progressive cognitive decline.

Another limit is connected to the difficulty to make a direct comparison of gut microbiota composition among studies: in particular, some studies underline a significant difference at phylum level, others at genus level, others at species level. This is probably related to the fact that there is not a uniform scale when comparing the taxonomy and the functions associated with the human microbiota. Finally, most of the existing studies included very small size sample of patients; so, we are still far from extending these results to a universal MCI population.

Although this microbiome analysis does not accurately identify a specific MCI profile, however modulating the relative deficit and overrepresented species by an appropriate supplementation is now becoming a topic of interest also in the context of MCI.

The few available studies about the use of probiotics in this setting show encouraging results, with a general improvement in cognitive assessment scales compared to the placebo group. However, they are limited by the small sample size and the limited follow-up duration. Additionally, different diagnostic criteria have been used for the diagnosis of MCI, as well as various scales for the cognitive assessment.

There are no studies comparing one type of supplementation to another, nor are there clear indications of how long the impact of taking probiotics for a limited period may last on the patients' microbiota. A longer follow-up of patients would certainly be interesting to evaluate this aspect as well. Particular attention should also be given to concurrent health conditions, considering that subjects with MCI usually have various comorbidities due to their adult/advanced age, they may generally take multiple medications and have different lifestyles or dietary habits. These factors put a different complexion on clinical studies but should be considered when facing such challenging issues.

This work has some limitations. Firstly, the narrative nature of the review may have affected the strength of the study in terms of objectivity, completeness of the literature search, and interpretation of findings. At the same time, it is the only useful type of article for providing a description of the state of the art on a topic with a scarcity of data, often heterogeneous and resulting from small sample size studies conducted in different areas of the world, with possible confounding factors also affecting the gut microbial composition.

Nonetheless, this is the first review aimed to describe the current state of knowledge of the relationship between microbiota and mild cognitive impairment, including an insight into the therapeutic possibilities in this field. We hope that our work could serve as an initial step in shedding more light on this topic, highlighting the limitations of the currently available literature data, and initiating a more systematic focus for future, more robust studies.

## Author contributions

AG: Conceptualization, Writing – original draft. AM: Conceptualization, Data curation, Writing – original draft. RL: Data curation, Supervision, Writing – review & editing. MC: Data curation, Supervision, Writing – review & editing. FI: Data curation, Writing – original draft. SC: Writing – original draft. FR: Data curation, Writing – original draft. FL: Supervision, Writing – review & editing. MM: Supervision, Writing – review & editing.
